# Type III Endoleak Leading to Aortic Rupture After Endovascular Repair

**DOI:** 10.7759/cureus.26895

**Published:** 2022-07-15

**Authors:** Yahia Suliemeh, Mahmoud Bouabane, Mohammad Deershamarkha, Adnan Benzirar, Omar El Mahi

**Affiliations:** 1 Department of Vascular Surgery, Faculty of Medicine, Mohamed First University/University Hospital Mohammed VI, Oujda, MAR

**Keywords:** evar, stent-graft, white classification, aortic rupture, type iii endoleak

## Abstract

Endovascular treatment of abdominal aortic aneurysm concerns the introduction of aortic endoprosthesis which aims to isolate aneurysm from the circulation. The leading complication of this technique is the endoleak which is defined by the persistence of blood flow within the aneurysm sac. The main risk is the rupture of the aorta which can jeopardize the vital prognosis of patient. White classification defines five types of endoleaks. Type III is secondary either to a disconnection between the components of the endoprosthesis (type IIIA) or to material damage (type IIIB). This type presents a particularly high risk of aortic rupture. Endovascular approach is the treatment of choice for this type of endoleaks through stent-grafts implantation. Type III endoleak may appear at any time after endovascular aortic aneurysm repair (EVAR). Although the frequency of endoleak after EVAR has been reduced after improvement and development of stent-graft systems, there are still many improvements and additions expected to improve the prognoses for patients after EVAR. Our case is an 80-year-old patient, who received an EVAR in 2012 for his abdominal aortic aneurysm, admitted to our vascular surgery department for a type III endoleak, for which he benefited from an endovascular treatment by placement of stent-grafts.

## Introduction

An abdominal aortic aneurysm (AAA) is a dilation or bulging in the wall of abdominal aorta due to degeneration that develops in a segment of this wall, the main risk of non-treatment is a rupture of the aortic wall causing potentially fatal bleeding. Choosing the best way to treat AAA depends on a combination of factors that include the form of the aneurysm, its location, and the general condition of the patient. It is possible to treat AAA by two methods; the first is conventional surgery consisting in opening the aneurysmal sac and placement of bypass graft. The second method is a minimally invasive procedure called endovascular aneurysm repair (EVAR) which involves isolating the aneurysm from circulation by inserting a stent-graft through small incision in the groin. This stent-graft would reinforce the weak segment of the aorta [1].

On September 7, 1990, Dr. Parodi teamed with vascular radiologist Palmaz, to perform the world’s first successful endovascular aneurysm repair (EVAR), and then in 1992, Dr. Parodi introduced the EVAR technique to vascular surgeons in Europe and the United States [2].

This method has a preference over the surgical method because it does not need a large incision, as well as can be performed under local anesthesia, and has a substantially shorter recovery than the conventional surgical approach. However, EVAR may have several complications; the most worrying one is the persistence of a continuous aneurysmal sac infusion, called endoleak that can appear at any time after the procedure of EVAR [1].

These endoleaks are classified into five types (I-V) according to their mechanism. In our case, the patient presented a type III endoleak secondary to a defect and disconnection of the components of aorto-bi-iliac endoprosthesis which was implanted nine years before the appearance of endoleak, this type is particularly dangerous due to the higher risk of rupture compared to the other types. For this reason, this type of endoleaks needs to be treated as quickly as possible. The best treatment of this type of endoleak is ensured by endovascular technique using a stent-graft that can cover the leak.

## Case presentation

An 80-years-old man was admitted to the emergency room at our hospital center for constipation and abdominal pain. The patient has a history of type 2 diabetes, arterial hypertension, and ischemic heart disease under treatment.

Medical history

In July 2012, the patient was admitted to the emergency department for constipation and pain in the lower two limbs evolving in a chronic mode, not improved by symptomatic treatment. The patient benefited from an abdominal CT angiography that showed an infrarenal AAA with the largest diameter at 6 cm and extended to 9.5 cm just before the bifurcation aortic. In addition, the CT angiography showed stenosis at the level of the right and left common iliac arteries for which the patient benefited from angioplasty and the placement of two stents in August 2012. Given the unavailability of endovascular materials needed to treat the AAA, the EVAR operation was reported. In September 2012, he benefited from an EVAR for his AAA in another institution with bilateral surgical femoral access and placement of aorto-bi-iliac endoprosthesis. A control CT angiography performed one month after surgery showed that the aneurysm is excluded from the blood flow by the aortic endoprosthesis which is in correct place without any complication. We have very little information about the evolution after the EVAR procedure because the patient was declared lost to sight and poorly followed.

At admission to our hospital center (November 2021), the physical examination revealed a para-umbilical beating mass and anemic syndrome (pallor, severe asthenia, and discolored conjunctivae), all evolving in an afebrile context with altered general condition.

**Figure 1:**
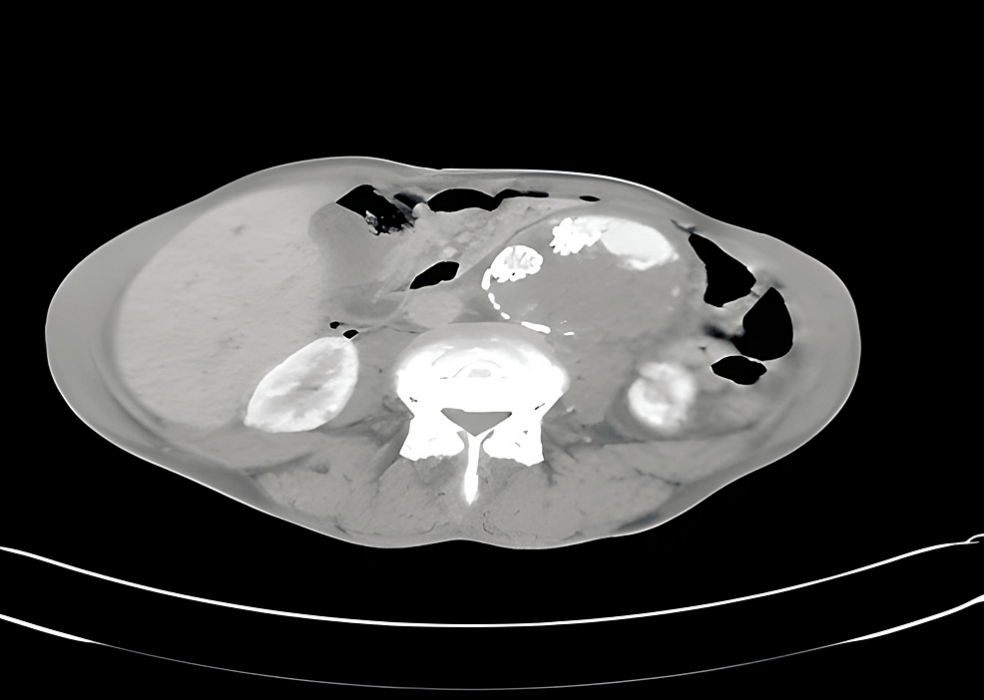
Axial slice of CT angiography demonstrating an endoleak with aortic rupture and hemoperitoneum.

**Figure 2:**
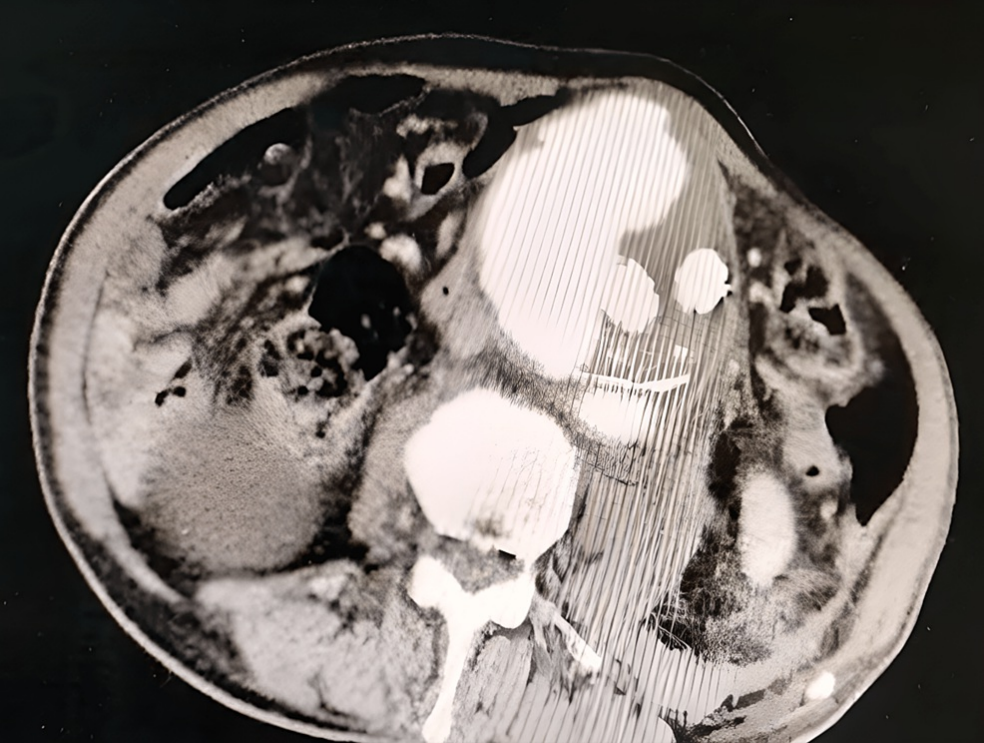
Axial slice of CT angiography demonstrating an endoleak with aortic rupture and hemoperitoneum.

A CT angiography showed a partially thrombosed fusiform aneurysm of the infrarenal abdominal aorta, measuring 89 mm on the axial plane and extending over 110 mm on the coronal plane which is the site of aorto-bi-iliac endoprosthesis, and also the presence of contrast product outside the prosthesis testifying of an endoleak with rupture and hemoperitoneum (Figures 1, 2).

**Figure 3:**
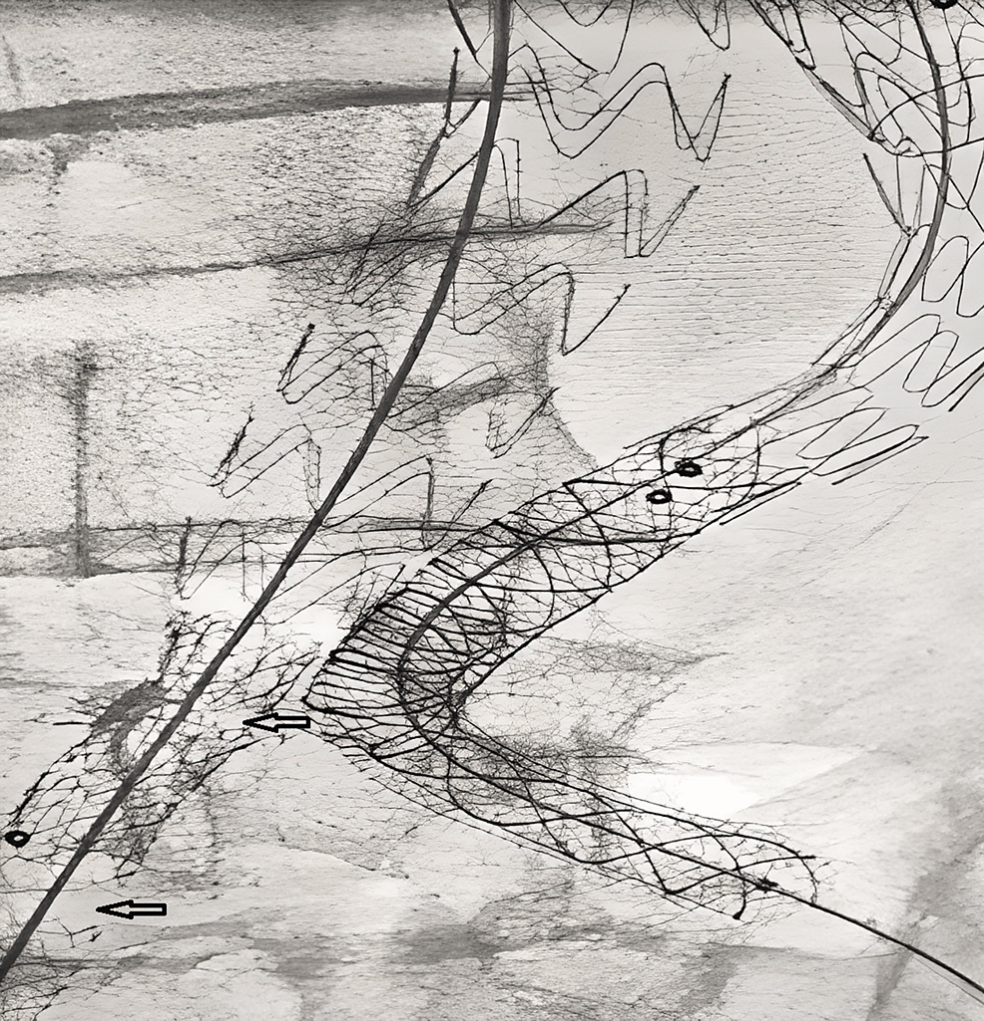
Arteriography showing a fabric defect (arrows) at the level of right limb of aorto-bi-iliac endoprosthesis and its extension.

**Figure 4:**
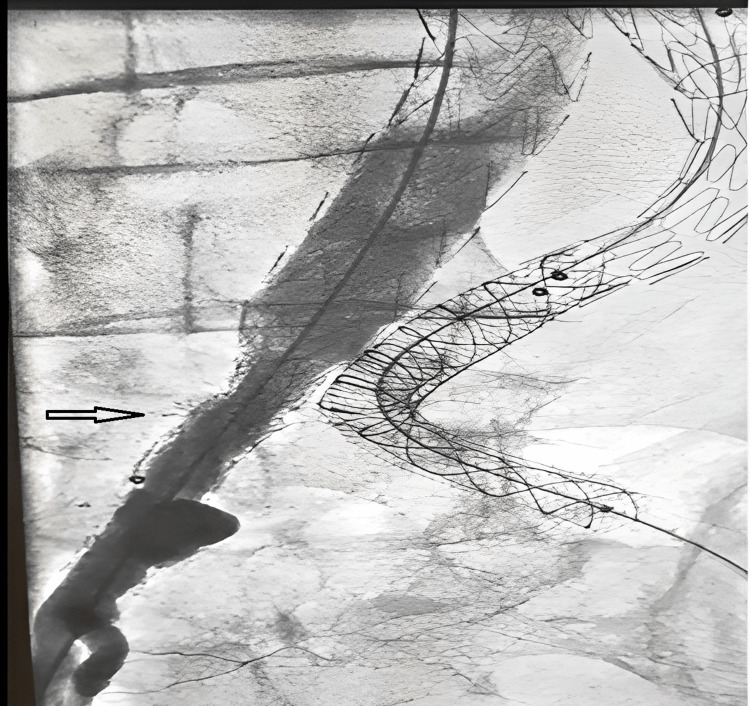
Arteriography showing a type IIIB endoleak (arrow) by fabric defect at the level of right limb of aorto-bi-iliac endoprosthesis.

**Figure 5:**
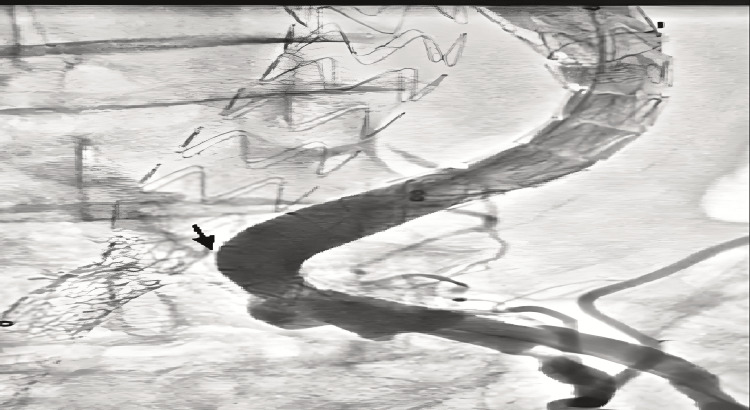
Arteriography showing a type IIIA endoleak (arrow) caused by the migration of the left limb extension of the aorto-bi-iliac endoprosthesis.

The diagnosis of type IIIB endoleak by fabric defect at the level of right limb of endoprosthesis was confirmed (Figures 3, 4). Conversely, the diagnosis of type IIIA endoleak by disconnection between the left limb and the stent-graft extension was retained (Figure 5).

**Figure 6:**
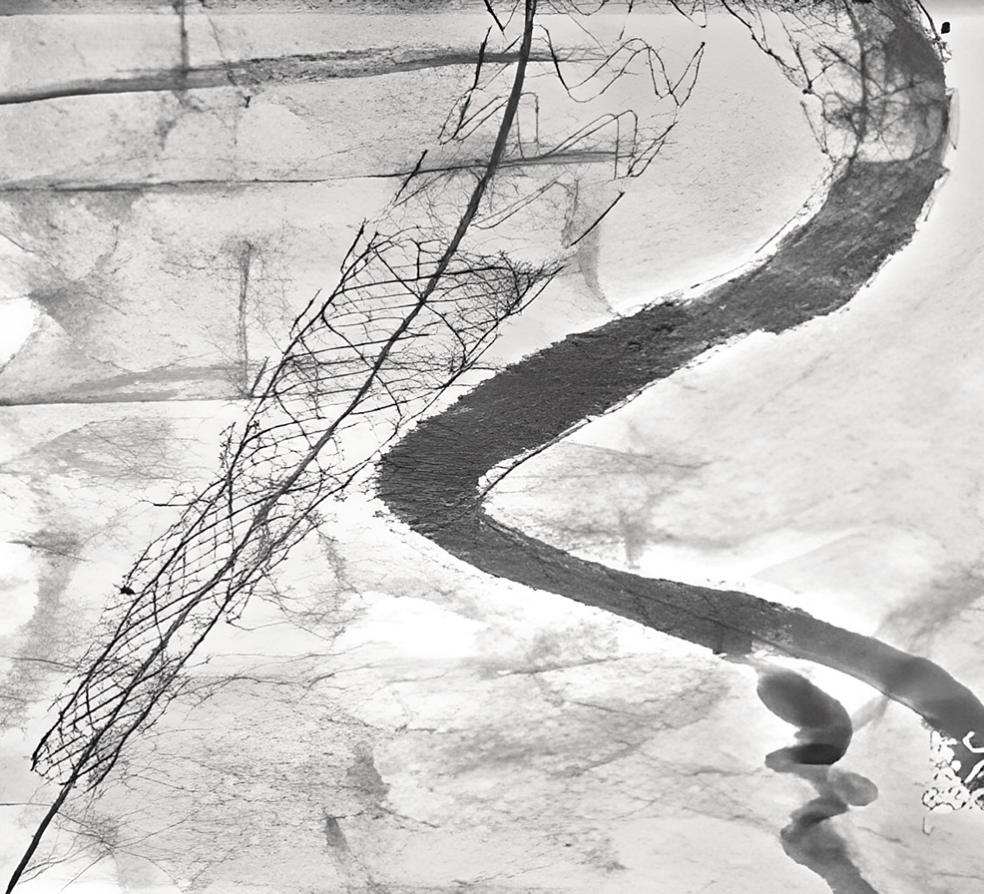
Arteriography after endovascular repair of endoleak at the left side. Disappearance of endoleak.

**Figure 7:**
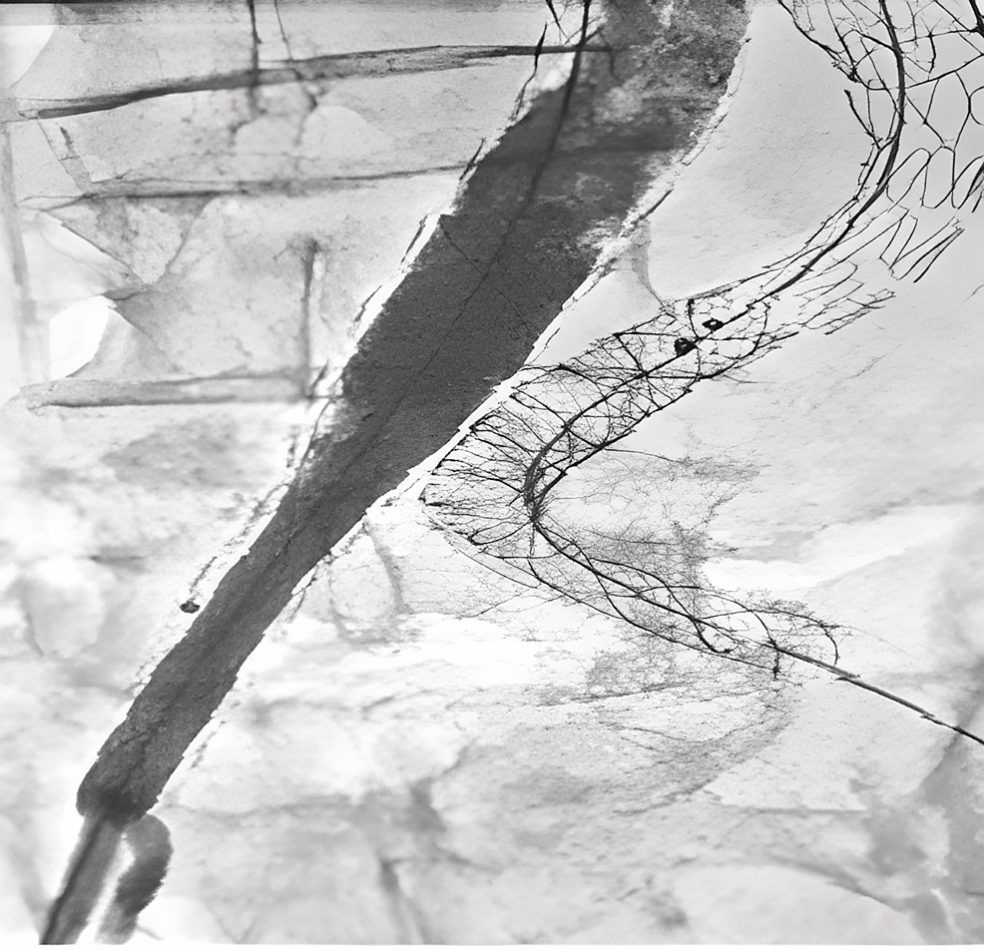
Arteriography after endovascular repair at the right side (placement of two stent-grafts). Disappearance of endoleak.

The patient has been treated urgently. He benefited from an endovascular treatment by placement of two stent-grafts (10 mm and 8 mm) covering the endoleak at the level of the left limb of the aorto-bi-iliac endoprosthesis (Figure 6), and placement of two stents (14 mm and 16 mm) covering the endoleak at the level of the right limb and between right limb and its extension (Figure 7).

The postoperative evolution was marked by the disappearance of the pulse of the abdominal mass, clinical and laboratory testing improvement (hemoglobin at 9 g/dL compared to 5 g/dL in admission knowing that the patient benefited from a transfusion with three units of red blood cells).

## Discussion

Definition and classification

Endoleaks are defined by the persistence of blood flow outside of the prosthesis and within the aneurysmal sac; they represent one of the main causes of endovascular procedure failure [3]. More than 20 years after the first procedure for EVAR, many issues related to this type of treatment remain unclear and ambiguous, and one of the most important issues is the incidence and importance of endoleaks. The first special classification of endoleaks after EVAR was made by White and Yu; this classification called the classification of White helped to understand the mechanism of the occurrence of these leaks by dividing these leaks into five types (types I-V endoleak) [4]. And there is an additional type of undetermined source in which the cause of the leak is unknown. The Society of Vascular Surgery developed a detailed explanation and description of these endoleaks [5].

**Figure 8:**
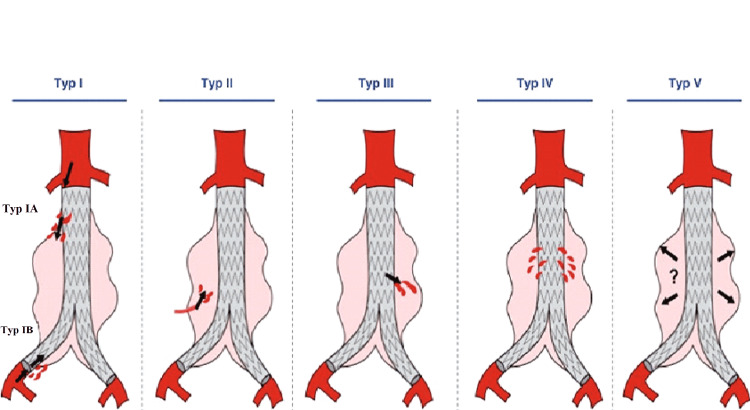
Classification of White - five types of endoleaks. The image is obtained from Bley and Roos (2019) [6].

The classification of White, commonly used, defines five types of endoleaks (Table 1 and Figure 8) according to their location and their origin (site of entry of blood into the aneurysmal sac) and independently of the type of prosthesis used [4,6]. This classification is essential because it identifies therapeutic management. Initially described for the infra-renal aorta, a similar classification of thoracic endoleaks is currently used. These endoleaks can also be classified based on the time they occur to primary endoleaks that occur in the first 30 days after EVAR, and secondary endoleaks that occur later.

**Table 1:** Classification of White (the mechanism responsible for each type of endoleaks). *Most commonly lumbar, mesenteric, or iliac collateral vessel leak. **Expansion of the aneurysm dimensions without visible endoleak.

Endoleak type	Source of endoleak
Type I	Attachment site leak: proximal (type IA) or distal (IB)
Type II	Aortic side branches*
Type III	Graft failure: midgraft hole, junctional leak, disconnect
Type IV	Graft wall porosity
Type V	Endotension**
Indeterminate	Not classifiable as types I-V endoleak

Frequency and pathology

The rate of incidence of all types of endoleaks combined is between 5% and 20% after endovascular treatment of thoracic and/or abdominal aortic by placement of stent-grafts [7,8]. The factors determining the incidence of an endoleak, whatever its type, are anatomical characteristics (particularly the diameter and the length of the upper and lower necks), the total distance to eliminate, the maximum aneurysm diameter, and the morphology, angulations, and calcifications of the aorta and iliac arteries [7].

The most contemporary and detailed definition of type III leakage is found in previously developed reporting standards by the Society for Vascular Surgery, this subgroup of endoleak is primarily due to mechanical failure of the graft (early component defect or late material fatigue) [6].

Type III endoleak is relatively unusual and only happens around 2% after an endovascular aneurysm repair (EVAR) [9]. The incidence of endoleak in the first and second-generation endografts is significant. In a retrospective study carried out by a group of specialized centers, which involved 965 patients, in order to understand the relationship between the effect of the stent-grafts generation on the rate of incidence of type III endoleak, the result showed a clear effect that the incidence of type III endoleak after EVAR by first or second-generation endografts was 12.7%, this rate has been reduced to 1.2% after using the third generation. At the same time, the study showed that the time of endoleak increased after using the third generation of endografts which was 5.92 years compared to 3.87 years after EVAR by first or second-generation [9]. From this study, we concluded that the development and improvement of stents have an excellent and effective impact on the incidence of type III endoleak, but so far there is no endograft system capable of avoiding type III endoleak in an absolute manner, and therefore the need for improvement and continuous change is a medical requirement.

Although they are rare, type III endoleaks should be taken seriously due to the rapid increase in pressure exerted by blood flow on the aneurysm wall and thus the increased risk of aortic rupture, which is almost nine times superior. Therefore, type III endoleak requires an early repair after imaging diagnosis [10].

Type III endoleak, in turn, is divided into two different sub-types according to the trigger mechanism. Type IIIA endoleak results from the separation or disconnection between the components of endoprosthesis (between the main body and the contralateral limb, iliac limb, and ipsilateral extension or between proximal cuff and the main body of endograft). Type IIIB is secondary to defects of the fabric of the endoprosthesis, like fabric ruptures or cracks.

The process of stent defect that causes this type of endoleak involves many hypotheses including fabric damage. One of these reasons is the displacement of the extremity of a stent, during the initial procedure by rubbing between a stent and calcified, tortuous, and twisty iliac arteries or by significant and severe neck angulation. Another reason that may lead to the damage of the fabric is the excessive pressure the balloon exerts on the stent [11]. Finally, late fabric defect can be the result of biologic degeneration.

Diagnostic methods

The diagnosis of endoleaks is essential since they are directly linked to the risks of revision, surgical conversion, and rupture. In fact, continued blood flow within sac aneurysmal results in maintaining positive intra-aneurysmal pressure and this would prevent size-reduction aneurysm; therefore, there is no protection against possible rupture [12,13]. In contrast, the most important criterion for the success of intravascular therapy is the observation of a reduction in the size of the aneurysmal sac [14].

In order to identify and reveal endoleaks or endotension phenomena, strict and systematic follow-up and control protocols must be followed after any endovascular procedure. The patient is supervised by repeated imaging examinations, carried out +/- 72 hours postoperatively, as well as at one, three, six, 12, and 24 months, then once a year in the absence of anomaly. CT angiography is still considered the most suitable and preferred imaging technique in the detection of endoleaks and must be rigorously integrated into patient supervision protocols [15].

The endoleak is diagnosed by the presence of hyperintense zone around the limits of the prosthesis, but included inside the aneurysmal sac, better perceived in the arterial or late phase, it is undetectable in the absence of contrast.

CT angiography can confirm type III endoleak by the presence of a disconnection between the components of stent graft. In the case of fabric defect, CT angiography can confirm the diagnosis of endoleak; however, there may be necessary to realize a catheter-directed angiography in order to determine the type and source of endoleak. [16,17].

The surveillance of stent-graft patients is very important, this is for determining the effectiveness of EVAR and the performance of stent-graft, this is commonly achieved with CT angiography realized at regular intervals [18]. The objectives of this continuous surveillance and periodic CT angiography are to assess the effectiveness of endovascular treatment (the response of the aneurysm sac) as well as the search for complications that can occur after EVAR both in the short and long term, the most important of which is the presence of endoleaks. Some also rely on the use of Doppler US for surveillance of stent-grafts [19].

The optimal treatment of endoleaks depends above all on their detection. This objective is particularly difficult for type V, not visible by conventional imaging (CTA) even though there is an increase in the diameter of the aneurysm. This suggests that the absence of visible endoleak may wrongly suggest the absence of endotension and that regular measurement of the diameter and/or volume of the aneurysmal sac must imperatively be part of the follow-up [20,21].

Management

The type III endoleaks are believed to be the most dangerous ones because of rapid and important repressurization of the aneurysm sac. this type of endoleaks is most often treated by endovascular method, this method involves covering the gap responsible for endoleak by placement of stent-graft extension between the components of the original endograft or through the fabric defect [22].

Treatment of early type of leakage involves additional expansion by using a suitable balloon or additional stent to achieve better interface. It is better to use a second covered stent-graft in order to ensure a connection between the components of the original endograft as well as to reconnect possible fabric ruptures [23].

In case of late-type III endoleak, the treatment involves repairing the defect by placing a covered stent through the gap resulting from the separation of the original endograft components or through the rupture of the fabric. In all cases, endovascular treatment is the most appropriate and best treatment, as well as complications associated with this type of treatment are less compared to surgical treatment.

The principal technical difficulty is catheterization of the second component, the difficulty of this step can be due to tortuosity and result in considerable movement and gap between the main body and the detached limb. In case of failure of retrograde cannulation from the groin, another attempt is possible to be performed by brachial artery access. A guidewire is progressed across a parent leading catheter via the entrance toward the sac. For retrieving the guidewire from the groin, we can use a snare device, after that, the guidewire may be replaced by a stiff rigid wire, by which a new stent can be installed so that it perfectly covers the source of endoleak [23]. In our case, the patient has benefited from the same endovascular technic mentioned above. Caution and throwing should be followed while crossing the wire, due to the risk of the wire passing between the interspaces of the stents of either element of original endograft’s components, this would make the stent placement process problematic.

There are other alternatives for endovascular treatment of type III endoleak. One of these alternatives is to utilize a new bifurcated stent-graft. If the principal body is too short, grafting of an aorto-uni-iliac stent and femoro-femoral bypass is an appropriate treatment. The indications of these alternatives are the proximity of the fabric defect to the flow diverter, also if the exact location of the defect is unknown, or when treating with several element disconnections [23].

## Conclusions

In conclusion, in our case, the diagnosis of type III endoleak was confirmed. It is possible that type III of endoleak can appear at any time after EVAR procedure. This type of endoleak is divided into two different sub-types depending on the trigger mechanism; sub-type IIIA endoleak which results from separation or disconnection between the original endograft components and sub-type IIIB endoleak secondary to a fabric defect. Endoleak type III has a high risk of aneurysmal sac rupture, accordingly, continuous follow-up of the patient after EVAR procedure by CTA or other imaging examinations constitutes an important step in the early diagnosis of endoleaks and in avoiding the complications of such endoleaks. Although the frequency of endoleak after EVAR has been reduced after improvement and development of stent-graft systems, there are still many improvements and additions expected to improve the prognoses for patients after EVAR. If type III endoleak is diagnosed, endovascular treatment is considered optimal for repairing damage by placement of stent-graft so as to cover the source of the endoleak; open surgical conversion can be a final option.
